# Circulating TNF Receptors 1 and 2 Predict Mortality in Patients with End-stage Renal Disease Undergoing Dialysis

**DOI:** 10.1038/srep43520

**Published:** 2017-03-03

**Authors:** Tomohito Gohda, Shuntaro Maruyama, Nozomu Kamei, Saori Yamaguchi, Terumi Shibata, Maki Murakoshi, Satoshi Horikoshi, Yasuhiko Tomino, Isao Ohsawa, Hiromichi Gotoh, Shuko Nojiri, Yusuke Suzuki

**Affiliations:** 1 Division of Nephrology, Department of Internal Medicine, Juntendo University Faculty of Medicine, Bunkyo-ku, Tokyo 113-8421, Japan; 2 Division of Endocrinology and Metabolism, Department of Internal Medicine, Hiroshima Red Cross Hospital and Atomic-bomb Survivors Hospital, Naka-ku, Hiroshima 730-8619, Japan; 3 Department of Internal Medicine, Saiyu Soka Hospital, Soka, Saitama 340-0041, Japan; 4 Clinical Research Support Center (JCRSC), Juntendo University, Bukyo-ku, Tokyo 113-8421, Japan.

## Abstract

Relatively high circulating levels of soluble tumor necrosis factor (TNF) receptors (TNFRs: TNFR1, TNFR2) have been associated with not only progression to end-stage renal disease but also mortality in patients with diabetes. It remains unknown whether elevated TNFR levels in haemodialysis patients are associated with mortality. We studied 319 patients receiving maintenance haemodialysis who were followed for a median of 53 months. Circulating markers of TNF pathway (TNFα and TNFRs) were measured with immunoassay. Strong positive correlations between TNFR1 and TNFR2 were observed (r = 0.81, *P* < 0.0001). During follow-up, 88 (27.6%) patients died of any cause (40 [45.5%] died of cardiovascular disease). In the Cox multivariate model, either TNFR but not TNFα remained a significant independent predictor of all-cause mortality (TNFR1: hazard ratio [HR] 2.34, 95% confidence interval [CI], 1.50–3.64; TNFR2: HR 2.13, 95% CI 1.38–3.29) after adjustment for age, prior cardiovascular disease, predialysis systolic blood pressure, and large systolic blood pressure decline during dialysis session. For cardiovascular mortality, significance was only observed in TNFR1 (TNFR1: HR 2.15, 95% CI 1.13–4.10). Elevated TNFRs levels were associated with the risk of cardiovascular and/or all-cause mortality independent of all relevant covariates in patients undergoing haemodialysis.

End-stage renal disease (ESRD) is associated with morbidity and mortality due to cardiovascular disease (CVD). However, the risk factors contributing to atherosclerosis, other than cholesterol, blood pressure, or smoking, remain to be clarified^1^,^2^,^3^. Recently, chronic low-grade inflammation, such as that due to tumor necrosis factor (TNF) system activity, has been shown to play an important role in the progression of not only kidney disease but also atherosclerotic disease^4^,^5^,^6^. TNF receptors (TNFRs: TNFR1, TNFR2) belong to the TNF receptor superfamily, a group of type I single transmembrane glycoproteins. Binding of TNFα to TNFR1 or TNFR2 activates signalling pathways that control inflammatory and immune responses, and apoptosis^7^,^8^. We previously demonstrated that the TNF-TNFR2 pathway is involved in the development and/or progression of diabetic nephropathy in the type 2 diabetic model of KK-A^y^ mice, and TNF inhibition with a soluble TNFR2 fusion protein (etanercept) improves albuminuria and renal tissue injury through its anti-inflammatory effect^9^. In humans, the results from the Joslin Kidney Study demonstrated that increased levels of circulating TNFRs are useful as very strong predictors of the progression of diabetic nephropathy to chronic kidney disease (CKD) stage 3 or ESRD^10^,^11^. Additionally, we reported that circulating levels of TNFRs are associated with albuminuria, estimated glomerular filtration rate (GFR), and severity of interstitial fibrosis in patients with IgA nephropathy^12^.

Although several studies have examined the association between inflammatory markers and mortality in haemodialysis patients^13^,^14^,^15^, little is known about whether elevated circulating TNFR levels in haemodialysis patients are associated with mortality^16^. Therefore, the purpose of this study was to determine TNFR levels and evaluate TNFRs as a risk factor for mortality in haemodialysis patients.

## Results

### Baseline characteristics of patients

**Figure 1:**
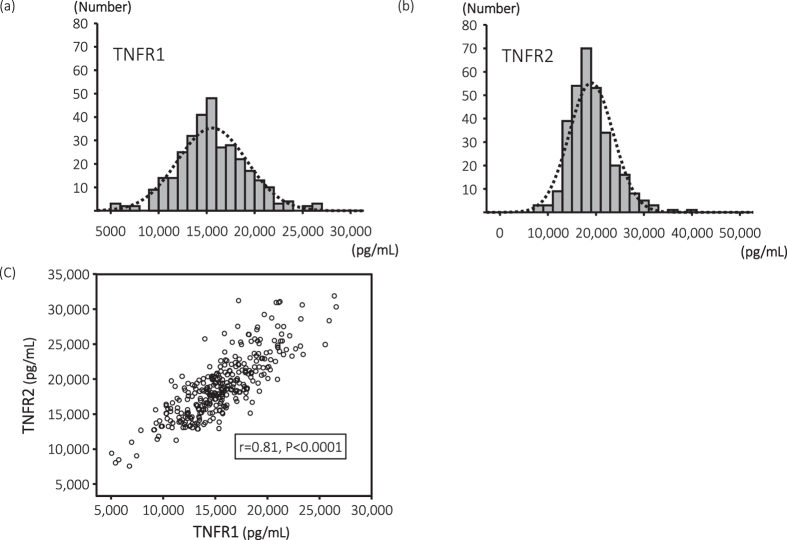
Histograms and scatter plots of TNFR1 and TNFR2. The median (25th, 75th percentiles) TNFR1 (Fig. 1a) and TNFR2 (Fig. 1b) levels were 15,383 (13,349, 17,888) and 18,504 (15,790, 21,335) pg/mL, respectively. Strong positive correlations between TNFR1 and TNFR2 were observed (Fig. 1c).

The mean (SD) age of the study population was 66 (12) years; 193 (60.8%) patients were men and 160 (50.2%) patients had diabetes. The median (25th, 75th percentiles) dialysis vintage was 59 (24–118) months. The median (25th, 75th percentiles) TNFα, TNFR1, and TNFR2 levels were 38 (33, 46), 15,383 (13,349, 17,888), and 18,504 (15,790, 21,335) pg/mL, respectively. Figure 1a and b show the histograms of TNFR1 and TNFR2.

The clinical characteristics of the 319 haemodialysis patients, stratified according to outcome—survivors (alive) and nonsurvivors (died)—are summarized in Table 1. On January 31, 2016, 231 of the 319 patients (72.4%) remained alive after a median follow-up of 53 months. The remaining 88 patients (27.6%) died. Of the 88 patients who died, 40 (45.5%) died of CVD. Patients in the nonsurvivors group were older and more likely to have had prior CVD; had a higher maximum change in systolic blood pressure (ΔSBP), maximum change in diastolic blood pressure (ΔDBP), and pulse pressure; and had lower DBP, serum albumin, and high-density lipoprotein (HDL)-cholesterol levels. In addition, the serum levels of TNFα, TNFR1, TNFR2, and high sensitivity C-reactive protein (hs-CRP) in patients in the nonsurvivors group were significantly higher than those in the survivors group.

### Correlation among TNFα, TNFR1, and TNFR2

Significant positive correlations among three markers were observed. Notably, the correlation coefficient between the two TNFRs was 0.81 (Fig. 1c). As shown in Table 2, the levels of both TNFRs were positively correlated with TNFα (TNFR1: r = 0.40, *P* < 0.0001; TNFR2: r = 0.60, *P* < 0.0001) and hs-CRP (TNFR1: r = 0.39, *P* < 0.0001; TNFR2: r = 0.41, *P* < 0.0001) levels, and negatively correlated with HDL-cholesterol and non-HDL cholesterol levels. The levels of both TNFRs were also positively associated with dialysis vintage, maximum ΔSBP, maximum ΔDBP, and corrected calcium levels. Furthermore, TNFR2 levels were associated with age, DBP, and prior CVD. We performed a multivariate regression analysis of contributing factors to explain the TNFR levels. The levels of TNFα, hs-CRP, HDL-cholesterol, and dialysis vintage were significantly associated with TNFR levels. Moreover, age and non-HDL-cholesterol level were significantly associated only with the TNFR2 level.

### Association of TNFR levels with outcome

**Figure 2:**
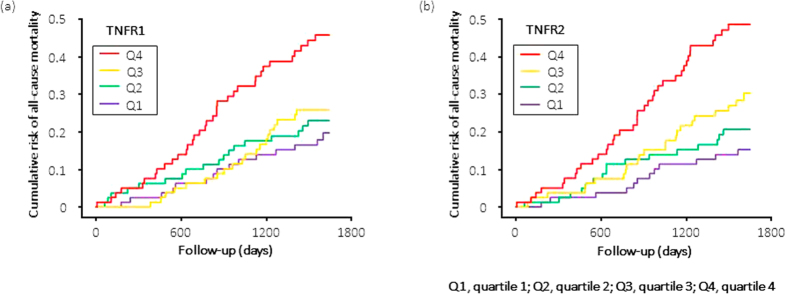
Cumulative risk for all-cause mortality in haemodialysis patients during 53 months of follow-up according to quartiles of circulating TNFR1 and TNFR2 at baseline. The cumulative risk for all-cause mortality steeply increased at a constant rate from the start of observation for patients in the highest quartile of TNFR1 (Fig. 2a) or TNFR2 (Fig. 2b).

**Figure 3:**
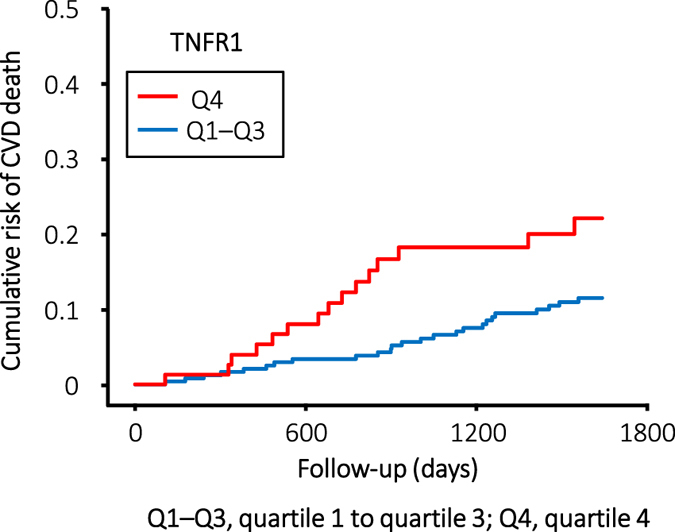
Cumulative risk for cardiovascular mortality in haemodialysis patients during 53 months of follow-up according to quartiles of circulating TNFR1 at baseline. The cumulative risk for cardiovascular mortality increased for patients in the highest quartile of TNFR1, but the event rate of cardiovascular mortality was relatively low in comparison with that of all-cause mortality.

To determine the effects of marker concentration on the temporal pattern of occurrence of all-cause and cardiovascular mortality, we plotted the cumulative risk for all-cause and cardiovascular mortality according to follow-up time and marker quartile. For patients in the highest quartile of TNFR1 or TNFR2, the cumulative risk for all-cause mortality steeply increased at a constant rate from the start of observation (Fig. 2). A similar pattern was observed for cardiovascular mortality; however, the event rate was low (Fig. 3).

Table 3 shows the results of univariate and multivariate Cox proportional analyses. Whereas many baseline clinical covariates showed an association with risk for all-cause mortality in univariate analysis, almost all of them became insignificant when analyzed together. Age, body mass index, SBP, maximum ΔSBP, HDL-cholesterol, and prior CVD remained significant in multivariate analysis with clinical predictors. Compared with the reference category of SBP (140–160 mmHg), the all-cause mortality was elevated for patients with SBP < 140 mmHg (hazard ratio [HR] 2.30, 95% confidence interval [CI] 1.30–4.07, *P* = 0.004) and >160 mmHg (HR 1.79, 95% CI 1.00–3.19, *P* = 0.049), indicating a U-shaped association between predialysis SBP and mortality. Next, we assessed the independent effect of each TNF marker on the risk of all-cause mortality by adding it to a Cox proportional hazard model of the influential clinical predictors. In this model, TNFα became insignificant; however, each of the TNFRs remained significant. The results for cardiovascular mortality are shown in Table 4.

### Predictive value of TNF-related biomarkers for all-cause mortality

To examine the clinical benefit of TNF-related biomarkers compared with hs-CRP as reliable predictors of all-cause mortality, we calculated the area under the receiver-operating characteristic (ROC) curve (AUC), the integrated discrimination improvement (IDI), and the net reclassification index (NRI). TNFR2 added a significant benefit for the prediction of all-cause mortality when measured together with hs-CRP (ΔAUC 0.13, *P* = 0.04). However, the addition of each TNF-related biomarker (TNFα and TNFRs) to the basic model consisting of age, prior CVD, SBP, maximum ΔSBP, and hs-CRP did not improve the AUC (Supplementary Table 1).

The addition of each TNFR to hs-CRP improved the prediction of all-cause mortality (TNFR1; IDI 0.014, *P* = 0.04, NRI 0.389, *P* < 0.01, TNFR2; IDI 0.041, *P* < 0.001, NRI 0.425, *P* < 0.001). When each TNFR, especially TNFR2, was added to the basic model, there was again an added benefit with respect to all-cause mortality compared with the basic model alone (TNFR1; IDI 0.008, *P* = 0.08, NRI 0.277, *P* = 0.03, TNFR2; IDI 0.022, *P* < 0.001, NRI 0.373, *P* < 0.01) (Supplementary Table 2).

### Subgroup analyses according to the baseline characteristics

**Figure 4:**
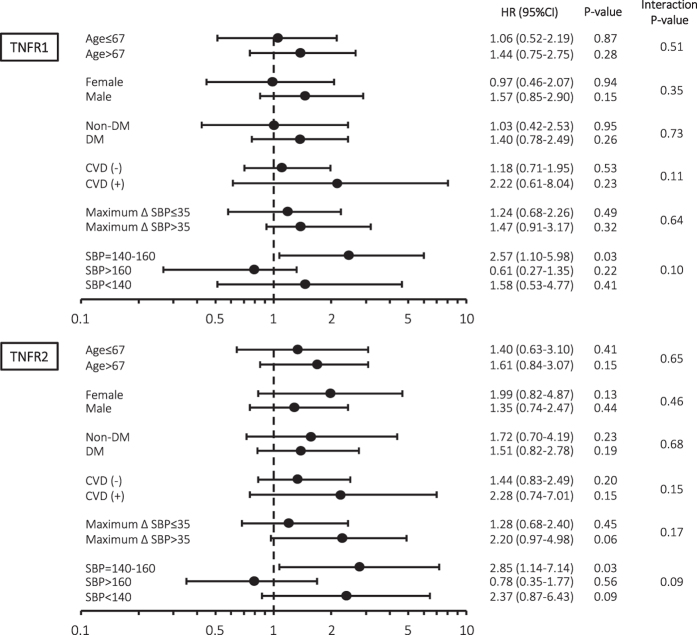
Subgroup analyses of risk of all-cause mortality according to the baseline characteristics and circulating TNFR1 or TNFR2 at baseline. After adjustments for age, history of CVD, maximum ΔSBP, and hs-CRP, TNFR1 and TNFR2 were found to be independent predictors of all-cause mortality in patients with a predialysis SBP of 140–160 mmHg.

Subgroup analyses were performed to confirm the predictive benefit of TNFRs in the different subgroups. After adjustments for age, prior CVD, maximum ΔSBP, and hs-CRP, each TNFR was found to be an independent predictor of all-cause mortality in patients with a predialysis SBP of 140–160 mmHg. No significant interactions were observed among the subgroups (Fig. 4).

## Discussion

The major finding of our study is that relatively high levels of circulating TNFRs were strongly associated with the risk of all-cause and/or cardiovascular mortality in haemodialysis patients. This association was independent of age, prior CVD, SBP, and maximum ΔSBP. Cardiovascular mortality was approximately 60% higher in patients in the highest quartile (Q4) of TNFR1 when compared with those in the other quartiles (Q1–Q3).

A strong correlation between the levels of TNFR1 and TNFR2 was observed even in patients undergoing haemodialysis, as well as in predialysis CKD patients or patients in a community-based setting; however, the levels in haemodialysis patients were much higher than those in patients with residual renal function^12^,^17^,^18^,^19^. Considering the wide range in the distribution of TNFR levels despite the little residual renal function and a basically similar renal function in haemodialysis patients, the circulating TNFR levels might be defined by some factors other than renal function. In fact, circulating TNFR levels were associated with dialysis vintage, dyslipidemia, and TNFα (inflammation) in multivariate regression analysis in the present study. However, we do not know why the correlation between the levels of TNFR1 and TNFR2 was very strong in spite of the difference in the signalling pathway after the binding of each receptor. Earlier functional studies in animal models indicated that the involvement of TNFR1 and TNFR2 in the pathogenesis of disease development and/or progression varies by types of renal disease^20^,^21^,^22^. Further studies are needed in order to understand the mechanism of TNFR1/TNFR2 secretion into the circulatory system.

CKD patients are often in a state of chronic inflammation due to the upregulation of proinflammatory cytokines^23^,^24^,^25^. Chronic inflammation may cause malnutrition and consequently atherosclerosis through vascular endothelial dysfunction and vascular calcification—referred to as malnutrition-inflammation-atherosclerosis syndrome^26^. TNFα is a central proinflammatory cytokine and, at the same time, has immune-regulatory functions. Binding of TNFα to TNFR1 or TNFR2 produces distinct signalling pathways that may promote tissue injury or induce protective responses^7^,^8^. To date, many investigators including us have demonstrated that the TNF pathway is involved in the pathogenesis of various types of renal diseases^9^,^27^,^28^,^29^, and that TNF-related biomarkers are also associated with the levels of albuminuria or GFR^18^,^19^. However, little is known about whether TNF-related biomarkers predict the prognosis of haemodialysis patients. Recently, Carlsson *et al*.^16^ reported that circulating TNFR levels did not predict mortality in 207 haemodialysis patients. In sharp contrast with their result, our results support previous findings of an association between relatively high circulating TNFR levels and mortality in various diseases, including rheumatoid arthritis, diabetic kidney disease, and even community-based disaease^6^,^30^,^31^,^32^,^33^. The conflicting findings between their study and our study might be due to the following reasons. First, as the ELISA kits used for TNFR measurement were different, it is difficult to make a simple comparison between these results. In fact, the median levels of circulating TNFRs in our study were much lower than those in their study (ours vs. Carlsson *et al*.: TNFR1, 15,383 vs. 17,680 pg/mL; TNFR2, 18,504 vs. 24,450 pg/mL). It is, however, reported that those levels might vary among racial and ethnic groups. For instance, the levels of circulating TNFRs in patients with type 2 diabetes and in American Indians seem to be higher than those in Caucasians or Asians, even in patients with comparable renal function levels^11^,^34^,^35^. Second, another factor other than TNFRs might be strongly involved in the mortality of Western haemodialysis patients because considerable international difference in mortality was observed in haemodialysis patients^36^.

There are some valid reasons to incorporate hs-CRP into measurements for prediction of mortality in routine clinical practice. hs-CRP not only has relatively high predictive value for mortality, but testing is also readily accessible in a typical dialysis unit, and more importantly, is inexpensive^37^,^38^,^39^. In the present study, we calculated AUC, NRI, and IDI to examine the clinical value of TNFR compared with hs-CRP as a reliable predictor of mortality. Although reclassification metrics such as NRI and IDI were statistically improved following incorporation of TNFR levels, especially that of TNFR2, the additive clinical value of each TNFR to CRP or the basic model might be limited when considering the AUC. However, the AUC is an overall measure of discrimination, because a specific diagnostic algorithm generally uses a specific diagnostic cut-off value^40^. The NRI and IDI should be considered when evaluating the usefulness of biomarkers, because the AUC can be insensitive to important changes in absolute risk, and one may overlook the value of biomarkers when using the AUC alone^41^,^42^. Given the results of AUC, NRI, and IDI, the predictive performance of TNFR2 seems to be somewhat superior to that of TNFR1. However, a Cox proportional hazards model revealed that the performance of each TNFR seems to be comparable, and determines whether variables can be characterized as either continuous or categorical. In any event, the measurement of TNFR is of importance in stratifying patients at risk for mortality because mortality rates for haemodialysis patients remain extraordinarily high.

Inaba *et al*.^2^ reported that both low and high BP are associated with all-cause mortality in 9134 Japanese haemodialysis patients of the Dialysis Outcomes and Practice Patterns Study cohort. Similar to their results, a U-shaped association between SBP and all-cause mortality was observed, with the lowest mortality for predialysis SBP 140–160 mmHg, in the present study. It is interesting to note that, in the subgroup analysis, elevated baseline TNFR levels are associated with all-cause mortality in patients with a relatively good prognosis whose predialysis SBP is 140–160 mmHg (TNFR1; HR [95% CI] 2.57 [1.10–5.98], *P* = 0.03, TNFR2; HR [95% CI] 2.85 [1.14–7.14], *P* = 0.03). Park *et al*.^43^ recently reported that modest declines in BP after haemodialysis are associated with mortality in a large-scale retrospective cohort study. In the present study, however, ΔSBP and ΔDBP did not predict mortality in haemodialysis patients. Nevertheless, predialysis low SBP and maximum ΔSBP were strongly associated with mortality, indicating that BP fluctuations during haemodialysis have a large impact on the mortality of haemodialysis patients.

The potential limitations of this study need to be acknowledged. First, as the patients of the present study were all Japanese ESRD patients in one haemodialysis unit, the associations between TNFR levels and mortality may not be applicable to other populations. Second, the cardiovascular and all-cause mortality rates in this study were lower than those reported in previous studies on Western dialysis patients^36^. We speculate that the difference in ethnicities might have contributed to these disparate results. Third, maximum ΔSBP and maximum ΔDBP were handled as covariates for statistical analysis; however, practically, we cannot capture the proper maximum and minimum BP during every dialysis session. To overcome the drawback of BP measurement timing, we averaged each BP value in 12 times dialysis sessions. Finally, especially given that unmeasured patient characteristics affect BP, the number of study patients is too small to conclude the optical BP level in haemodialysis patients. Despite these limitations, to our knowledge, the present study is the first to investigate the association of baseline TNFR concentrations with cardiovascular and all-cause mortalities in ethnically homogeneous Japanese haemodialysis patients.

In conclusion, the risk of cardiovascular and/or all-cause mortality in haemodialysis patients was strongly associated with elevated concentrations of circulating TNFRs during a median follow-up of 53 months. Validation of these results in other studies may allow the use of TNFRs as practical serum markers of mortality in haemodialysis patients. If our results are true, then haemodialysis patients whose circulating TNFR levels are relatively high need to be monitored closely.

## Materials and Methods

### Study design

This prospective cohort study was carried out at Saiyu Soka Hospital located in the Tokyo metropolitan area. Study participants were recruited from August 1 to December 31, 2011. Initially, all patients (n = 354) undergoing haemodialysis were screened. Patients with advanced dementia based on medical review were excluded. Hospitalization within 2 months and receipt of haemodialysis for <1 month were set as temporary exclusion criteria. Finally, 319 clinically stable patients with baseline serum data available were followed until January 31, 2016. This study was approved by the ethics committee of Saiyu Soka Hospital. Informed consent was obtained from all patients, and this study complies with the Declaration of Helsinki. Serum samples were obtained before the start of haemodialysis at the beginning of the week and were stored at −80 °C until use.

In all patients, a thorough medical history was taken at the time of study enrollment by a trained physician. Prior CVD was defined as a medical history and clinical findings of coronary artery, cerebrovascular, and peripheral vascular diseases. Supine blood pressure (BP) measurements were performed at least seven times, including pre- and postdialysis BP, during every dialysis session by using automatically inflated cuffs and a digital monitor attached to each haemodialysis machine. A total of 12 times (11 times before recruitment and at the time of recruitment) average pre- and post-BP values were used for the analysis. Changes (Delta [Δ]) in systolic BP (SBP) and diastolic BP (DBP) were defined as predialysis BP minus postdialysis BP during every dialysis session. Maximum ΔSBP was defined as maximum SBP minus minimum SBP, and maximum ΔDBP was defined as maximum DBP minus minimum DBP during every dialysis session. Pulse pressure was defined as predialysis SBP minus predialysis DBP during every dialysis session. Twelve times average changes in predialysis SBP and DBP, maximum SBP and DBP, minimum SBP and DBP, ΔSBP and ΔDBP, maximum ΔSBP and ΔDBP, and pulse pressure were also calculated for the analysis.

The primary outcome measures were cardiovascular mortality and all-cause mortality from the time of sampling in the study. Cardiovascular mortality included fatal myocardial infarction, stroke, lethal arrhythmia, congestive heart failure, and sudden death.

### Laboratory measurement

According to manufacturer protocols, we used enzyme-linked immunosorbent assay (ELISA) to measure TNFR1, TNFR2 (cat. no. DRT100, DRT200; R&D Systems, Minneapolis, MN, USA) and total TNFα (cat. no. KAC1751; Invitrogen, Carlsbad, CA, USA). Two internal serum controls were included in each assay to estimate the inter-assay coefficient of variation. The inter-assay coefficient of variation for TNFα and TNFRs was very good and was around 3.0–4.0% (TNFα, 4.0%; TNFR1, 3.0%; TNFR2, 3.4%). High-sensitivity C-reactive protein (hs-CRP) was measured through nephelometry, a latex particle-enhanced immunoassay (N Latex CRP II; Dabe Behring, Tokyo, Japan).

### Statistical analyses

All variables are expressed as percentages for categorical data, and as means ± standard deviation (SD) or median and interquartile ranges for continuous data with and without a normal distribution, respectively. For analytical purposes, patients were stratified according to their quartile of TNFR levels. Differences between groups were checked with a t-test or Kruskal-Wallis and Mann-Whitney depending on the distribution (normal or skewed, respectively). Spearman correlation analysis was used to assess the associations among inflammatory biomarkers. Data were obtained from local electronic and paper records. Univariate and multivariate Cox proportional hazards regression analysis was used to examine the association of baseline variables with cardiovascular and all-cause mortality. The variables associated with the outcome at *P* < 0.05 on the basis of the univariate models were introduced in the multivariate models. Since the correlation coefficient between TNFR1 and TNFR2 was very strong, each TNFR was included the model separately to avoid multicollinearity. The contributions of each biomarker to the prediction of all-cause mortality were calculated according to AUC, IDI, and NRI. *P* < 0.05 was considered statistically significant. Statistical analysis was performed using SPSS 23.0 (IBM Inc., Armonk, NY, USA) or SAS 9.4 (SAS Institute, Cary, NC) software.

## Additional Information

**How to cite this article:** Gohda, T. *et al*. Circulating TNF Receptors 1 and 2 Predict Mortality in Patients with End-stage Renal Disease Undergoing Dialysis. *Sci. Rep.* **7**, 43520; doi: 10.1038/srep43520 (2017).

**Publisher's note:** Springer Nature remains neutral with regard to jurisdictional claims in published maps and institutional affiliations.

## Supplementary Material

Supplementary Information

## Figures and Tables

**Table 1:** Baseline characteristics of haemodialysis patients according to outcome.

	Survivors (n = 231)	Non-survivors (n = 88)	*P*
**Baseline characteristics**			
Age (yrs)	64±11	70±11	<0.0001
Male (%)	135 (58.4%)	59 (67.0%)	0.16
Diabetes mellitus (%)	112 (48.5%)	48 (54.5%)	0.33
Prior CVD (%)	51 (22.1%)	38 (43.2%)	<0.0001
Dialysis vintage (month)	79±87	92±84	0.25
Kt/V	1.3±0.3	1.3±0.3	0.54
BMI (kg/m^2^)	21.4±3.3	20.7±3.5	0.08
SBP (mmHg)	150±19	151±24	0.49
DBP (mmHg)	79±11	76±11	0.04
ΔSBP (mmHg)	1.9±16.6	3.2±20.0	0.57
ΔDBP (mmHg)	−0.1±7.1	0.6±8.5	0.55
Maximum ΔSBP (mmHg)	37±15	44±19	<0.001
Maximum ΔDBP (mmHg)	12±8	15±9	0.002
Pulse pressure (mmHg)	71±15	76±19	0.03
HDL-cholesterol (mg/dL)	44±13	38±13	<0.001
Non HDL-cholesterol (mg/dL)	112±35	113±36	0.79
Hemoglobin A1c (%)	6.1±1.1	6.0±1.3	0.66
Albumin (g/dL)	3.5±0.3	3.4±0.5	0.04
Corrected Ca (mg/dL)	9.6±0.7	9.7±0.9	0.16
Phosphate (mg/dL)	5.4±1.6	5.5±1.8	0.73
**Inflammatory markers**			
hs-CRP (mg/L)	0.8 (0.3, 2.7)	1.9 (0.5, 5.3)	0.003
TNFα (pg/mL)	37 (32, 45)	42 (35, 47)	0.046
TNFR1 (pg/mL)	15078 (13141, 17073)	17056 (14483, 18982)	0.003
TNFR2 (pg/mL)	18044 (15380, 20385)	20614 (17753, 24499)	<0.0001

Data are mean ± SD, median (quartiles), or %. CVD, cardiovascular disease; BMI, body mass index; SBP, systolic blood pressure; DBP, diastolic blood pressure; maximum ΔSBP, maximum SBP minus minimum SBP during every dialysis session; maximum ΔDBP, maximum DBP minus minimum DBP during hemodialysis every dialysis session; ΔSBP, predialysis SBP minus postdialysis SBP; ΔDBP, predialysis DBP minus postdialysis DBP; HDL, high-density lipoprotein; hs-CRP, high-sensitivity C-reactive protein; TNF, tumor necrosis factor; TNFR, TNF receptor.

**Table 2:** Simple and stepwise multiple regression analysis of variables which associated with TNFRs in haemodialysis patients.

Parameter	TNFR1		TNFR2	
	Model 1	Model 2	Model 1	Model 2
	Correlation coefficients	Standardized coefficients	Correlation coefficients	Standardized coefficients
	r	β	r	β
Age	0.10		0.19^†^	0.11^**^
Sex	−0.11		−0.08	
BMI	−0.12^*^		−0.07	
Dialysis vintage	0.36^††^	0.26^††^	0.30^††^	0.21^††^
Diabetes mellitus	0.02		0.08	
Prior CVD	0.11		0.19^†^	
SBP	0.03		−0.02	
DBP	−0.03		−0.13^*^	
Maximum ΔSBP	0.17^**^		0.17^**^	
Maximum ΔDBP	0.18^**^		0.15^**^	
Albumin	−0.07		−0.20^††^	
HDL-cholesterol	−0.21^††^	−0.16^†^	−0.31^††^	−0.11^**^
Non HDL-choleserol	−0.19^†^		−0.11^*^	−0.09^*^
Corrected Ca	0.12^*^		0.19^†^	
Phosphate	0.06		0.09	
TNFα	0.40^††^	0.30^††^	0.60^††^	0.49^††^
hs-CRP	0.39^††^	0.25^††^	0.41^††^	0.20^††^
	R = 0.56		R = 0.71	
	Adusted R^2^ = 0.32		Adusted R^2^ = 0.50	

Abbreviations used in this table are the same as in Table 1. ^*^P < 0.05, ^**^P < 0.01, ^†^P < 0.001, ^††^P < 0.0001.

**Table 3:** Cox proportional hazard analysis of risk for all-cause mortality in haemodialysis patients according to clinical predictors and circulating TNF related markers in univariate and multivariate models.

Baseline Characteristics			Multivariate model			
	Univariate model		Clinical predictors only		Clinical predictors and markers^a^	
	HR (95% CI)	*P*	HR (95% CI)	*P*	HR (95% CI)	*P*
**Clinical predictors (Units of increase)**						
Female gender	0.71 (0.46–1.11)	0.14				
Age (10 yrs)	1.57 (1.26–1.95)	<0.0001	1.34 (1.07–1.71)	0.009	1.39 (1.12–1.73)	0.004
Body mass index (1 kg/m^2^)	0.93 (0.87–0.998)	0.045	0.91 (0.85–0.98)	0.01		
Dialysis vintage (1 yr)	1.01 (0.99–1.04)	0.24				
Diabetes mellitus (0 = no; 1 = yes)	1.21 (0.80–1.84)	0.37				
Prior CVD (0 = no; 1 = yes)	2.23 (1.46–3.40)	<0.0001	1.66 (1.07–2.58)	0.02	1.84 (1.19–2.84)	0.006
HDL-cholesterol (1 mg/dL)	0.97 (0.95–0.99)	<0.001	0.97 (0.95–0.99)	0.004		
Albumin (1 g/dL)	0.42 (0.25–0.69)	<0.001				
hs-CRP (1 mg/L)	1.20 (1.06–1.35)	0.003				
SBP (10 mmHg)	1.00 (0.99–1.02)	0.47				
SBP [mmHg]						
140–160 (ref.) vs. <140	2.03 (1.17–3.54)	0.01	2.30 (1.30–4.07)	0.004	2.07 (1.16–3.68)	0.01
140–160 (ref.) vs. > 160	2.09 (1.21–3.62)	0.008	1.79 (1.00–3.19)	0.049	1.56 (0.87–2.78)	0.13
Maximum SBP (10 mmHg)	1.06 (0.94–1.19)	0.29				
Maximum SBP [mmHg]						
155–165 (ref.) vs. <155	2.20 (0.97–4.95)	0.06				
155–165 (ref.) vs. > 165	2.72 (1.23–6.02)	0.01				
Minimum SBP (10 mmHg)	0.87 (0.77–0.98)	0.02				
DBP (per 10 mmHg)	0.81 (0.67–0.99)	0.03				
DBP [mmHg]						
<75 (ref.) vs. ≥75	0.57 (0.37–0.86)	0.008				
Maximum DBP (10 mmHg)	0.82 (0.66–1.02)	0.07				
Minmum DBP (10 mmHg)	0.67 (0.55–0.82)	<0.0001				
Δ SBP (10 mmHg)	1.03 (0.92–1.16)	0.61				
Δ DBP (10 mmHg)	0.91 (0.68–1.23)	0.56				
Maximum Δ SBP (10 mmHg)	1.27 (1.13–1.43)	<0.0001	1.23 (1.08–1.42)	<0.001	1.23 (1.10–1.39)	<0.0001
Maximum Δ DBP (10 mmHg)	1.39 (1.12–1.73)	0.003				
Pulse pressure (10 mmHg)	1.17 (1.02–1.35)	0.02				
Pulse pressure [mmHg]						
<85 (ref.) vs. ≥85	1.77 (1.13–2.76)	0.01				
**Circulating TNF markers: Categorical variable, Q1–3 (ref.) vs. Q4**						
TNFα	1.64 (1.05–2.56)	0.03			1.46 (0.93–2.30)	0.10
TNFR1	2.42 (1.58–3.70)	<0.0001			2.34 (1.50–3.64)	<0.0001
TNFR2	2.70 (1.77–4.13)	<0.0001			2.13 (1.38–3.29)	<0.0001
**Continuous variable**						
TNFα (1SD = 0.29)	1.29 (1.01–1.49)	0.046			1.04 (0.82–1.31)	0.76
TNFR1 (1SD = 0.25)	1.45 (1.15–1.84)	0.002			1.17 (0.91–1.51)	0.22
TNFR2 (1SD = 0.24)	1.60 (1.32–1.95)	<0.0001			1.28 (1.01–1.61)	0.04

^a^The effect of each TNF marker was examined separately while controlling for clinical predictors. Hazard ratios for clinical predictors are from the multivariate model with TNFR1. Q, quartile; Abbreviations used in this table are the same as in Table 1.

**Table 4:** Cox proportional hazard analysis of risk for cardiovascular mortality in haemodialysis patients according to clinical predictors and circulating TNF related markers in univariate and multivariate models.

Baseline Characteristics	Univariate model		Multivariate model^a^	
	HR (95% CI)	*P*	HR (95% CI)	*P*
**Clinical predictors (Units of increase)**				
Female gender	0.31 (0.14–0.70)	0.005	0.36 (0.16–0.81)	<0.01
Age (10 yrs)	1.28 (0.96–1.72)	0.10		
Body mass index (1 kg/m^2^)	0.98 (0.89–1.08)	0.71		
SBP (10 mmHg)	0.88 (0.75–1.03)	0.12		
SBP [mmHg]				
140–160 (ref.) vs. <140	2.45 (1.10–5.45)	0.03		
140–160 (ref.) vs. > 160	1.73 (0.74–4.04)	0.21		
DBP (10 mmHg)	0.80 (0.60–1.08)	0.14		
DBP [mmHg]				
<80 (ref.) vs. ≥80	0.48 (0.24–0.99)	0.045		
Prior CVD (0 = no; 1 = yes)	3.97 (2.12–7.43)	<0.0001	3.86 (2.05–7.25)	<0.0001
Albumin (1 g/dL)	0.38 (0.18–0.81)	0.01		
hs-CRP (1 mg/L)	1.24 (1.04–1.48)	0.02		
**Circulating TNF markers: Categorical variable Q1–3 (ref.) vs. Q4**				
TNFα	1.43 (0.73–2.82)	0.30	0.33 (0.15–0.75)	0.97
TNFR1	2.18 (1.15–4.15)	0.02	2.15 (1.13–4.10)	0.02
TNFR2	2.00 (1.04–3.83)	0.04	1.54 (0.80–2.99)	0.20
**Continuous variable**				
TNFα (1SD = 0.29)	1.21 (0.90–1.63)	0.20	1.10 (0.80–1.50)	0.57
TNFR1 (1SD = 0.25)	1.31 (0.94–1.83)	0.12	1.19 (0.84–1.70)	0.33
TNFR2 (1SD = 0.24)	1.50 (1.10–2.05)	<0.01	1.33 (0.97–1.82)	0.09

^a^The effect of each TNF marker was examined separately while controlling for clinical predictors. Hazard ratios for clinical predictors are from the multivariate model with TNFR1. Abbreviations used in this table are the same as in Table 1.
