# Supplementary material for: Circulating TNF Receptors 1 and 2 Predict Mortality in Patients with End-stage Renal Disease Undergoing Dialysis
Source: Sci Rep. 2017 Mar 3;7:43520. doi: 10.1038/srep43520 (PMC5335256; doi:10.1038/srep43520)
Supplement: Supplementary Information [file srep43520-s1.pdf]

Circulating TNF Receptors 1 and 2 Predict Mortality in Patients with End-stage Renal Disease Undergoing Dialysis

Tomohito Gohda, MD, PhD[1], Shuntaro Maruyama, MD, PhD[1], Nozomu Kamei, MD, PhD[2], Saori Yamaguchi, MD, PhD[1], Terumi Shibata[1], Maki Murakoshi, MD, PhD[1], Satoshi Horikoshi, MD, PhD[1], Yasuhiko Tomino, MD, PhD[1], Isao Ohsawa, MD, PhD[3], Hiromichi Gotoh, MD, PhD[3], Shuko Nojiri, PhD[4], Yusuke Suzuki, MD, PhD[1]

[1] Division of Nephrology, Department of Internal Medicine, Juntendo University Faculty of Medicine

[2] Division of Endocrinology and Metabolism, Department of Internal Medicine, Hiroshima Red Cross Hospital and Atomic-bomb Survivors Hospital

[3] Department of Internal Medicine, Saiyu Soka Hospital

[4] Clinical Research Support Center (JCRSC), Juntendo University

Supplementary Table 1. TNF-related biomarker added predictive benefit for prediction of all-cause mortality in hemodialysis patients -ROC curve analysis-

	AUC	Difference in AUC (95%CI)	*P*
hs-CRP	0.613		
TNFα & hs-CRP vs. hs-CRP	0.637	0.055 (-0.001, 0.055)	0.13
TNFR1 & hs-CRP vs. hs-CRP	0.632	0.064 (-0.027, 0.064)	0.43
TNFR2 & hs-CRP vs. hs-CRP	0.678	0.126 (0.003, 0.126)	0.04
Basic model[a]	0.733		
Basic model + TNFα vs. Basic model	0.733	0.005 (-0.006, 0.005)	0.91
Basic model + TNFR1 vs. Basic model	0.736	0.019 (-0.013, 0.019)	0.71
Basic model + TNFR2 vs. Basic model	0.744	0.034 (-0.014, 0.034)	0.40

ROC, receiver-operating characteristic; AUC, areas under the ROC

[a]Basic model: age, previous history of CVD, SBP (categorical), maximum ΔSBP, and hs-CRP

Abbreviations used in this table are the same as in Table 1.

Supplementary Table 2. TNF-related biomarker added predictive benefit for prediction of all-cause mortality in hemodialysis patients -IDI and NRI analysis-

	**IDI (95%CI)**	**Relative IDI**	***P***	**NRI (95%CI)**	***P***
TNFα & hs-CRP vs. hs-CRP	0.004 (-0.003, 0.012)	0.160	0.19	0.173 (-0.071, 0.418)	0.17
TNFR1 & hs-CRP vs. hs-CRP	0.014 (0.001, 0.027)	0.509	0.04	0.389 (0.154, 0.623)	0.002
TNFR2 & hs-CRP vs. hs-CRP	0.041 (0.018, 0.064)	1.487	<0.001	0.425 (0.185, 0.665)	<0.001
TNFα & Basic model vs. Basic model[a]	0.001 (-0.002, 0.004)	0.005	0.66	0.077 (-0.168, 0.322)	0.54
TNFR1 & Basic model vs. Basic model	0.008 (-0.001, 0.018)	0.06	0.08	0.277 (0.038, 0.516)	0.03
TNFR2 & Basic model vs. Basic model	0.022 (0.006, 0.039)	0.165	<0.01	0.373 (0.133, 0.614)	<0.01

IDI, integrated discrimination improvement; NRI, net reclassification index

[a]Basic model: age, previous history of CVD, SBP (categorical), maximum ΔSBP, hs-CRP

Abbreviations used in this table are the same as in Table 1.
